# Education in personalized medicine in Czech Republic

**DOI:** 10.1186/1878-5085-5-S1-A19

**Published:** 2014-02-11

**Authors:** Marie Karlikova, Ondrej Topolcan, Jiri Polivka Jr, Sarka Svobodova, Martin Pesta, Jiri Polivka, Judita Kinkorova, Radka Fuchsova

**Affiliations:** 1 Faculty of Medicine in Pilsen, Charles University in Prague, Czech Republic; 2 Biomedical Centre, Faculty of Medicine in Pilsen, Charles University in Prague, Czech Republic; 3 University Hospital in Pilsen, Czech Republic; 4 Technology Centre Academy of Sciences, Czech Republic

## 

Personalized medicine is a new philosophy in the health care. It consists in the application of innovative diagnostic methods and biotechnologies to the prediction of human pathologies and in the development of prevention and individual therapy-planning. The principal tool of personalized medicine is the application of new methods, mainly those of molecular biology (abbreviated as –omics methods) into all areas of medicine in order to comply with two principal goals: 1/ optimization of early diagnostics, prediction and prognosis, and 2/ rigorous individualization of therapy-planning as well as an individual approach to the patient. Final effect should be a completely new way to the prevention assurance. Actual knowledge of the principles of personalized medicine among clinicians and therefore their application are still low.

## Technological approaches

Our first approach was to find out the level of knowledge among different population groups by means of a surway. The results confirmed the above mentioned statement. We have initiated the education of personalized medicine which is targeted at students of bachelor and master programs as well as at post graduate students, clinicians, academicians and researchers. The education is provided by the means of in-room teaching as well as e-learning and internet resources. Our goal is to provide the teaching in cooperation with national and international medical associations.

## Results interpretation

In 2011 we organized a two-day opening conference “Personalised medicine – from bench to bed”. The content of lectures was various, from basics and overall perspectives of personalized medicine and -omics methods to case-studies as examples of personalized medicine in practice. Lecturers were both local and foreign, including members of EPMA. For two years we have been involved in the research and education concerning different issues of molecular biology, prevention and personalized medicine, in the frame of the EU project “Molecular genetics and personalized medicine” (http://www.molekgen.eu). Beside that, we have organized educational courses and workshops focused on biomarkers and their assessment, clinical importance and interpretation, in the frame of 3 projects supported by EU. The outcomes of our activities, both research and educative, have been presented at several local and international congresses.

## Outlook and expert recommendations

Our immediate goal is to establish the teaching of personalized medicine as a facultative course for students at the Faculty of Medicine and the Faculty of Health Care Studies in Pilsen. Final goal is to establish an independent mandatory course which should be taught at the Faculties of Medicine through the Czech Republic.

Supported by projects ED2.1.00/03.0040, ED1.07/2.3.00/09.0182, ED1.07/2.3.00/09.0142 and ED1.07/2.2.00/15.0046 from European Regional Development Fund and by Ministry of Health, Czech Republic - conceptual development of research organization (Faculty Hospital Pilsen - FNPl, 00669806).

